# Cerebrospinal fluid culture-positive bacterial meningitis increases the risk for neurologic damage among neonates

**DOI:** 10.1080/07853890.2021.2004318

**Published:** 2021-11-17

**Authors:** Huawei Wang, Xueping Zhu

**Affiliations:** Department of Neonatology, Children's Hospital of Soochow University, Suzhou City, Jiangsu Province, China

**Keywords:** Neonate, bacterial meningitis, cerebrospinal fluid culture, risk, neurologic damage

## Abstract

**Objective:**

This study aimed to compare the clinical features and outcomes of neonatal bacterial meningitis (NBM) between patients with positive and negative cerebrospinal fluid (CSF) cultures and determine the risk factors for CSF culture-positive NBM.

**Methods:**

We retrospectively reviewed the medical records of all patients with NBM. Perinatal clinical data, laboratory results, and cranial radiographs were obtained.

**Results:**

Among the 186 neonates who met the inclusion criteria. The risk factors for positive CSF culture results were analysed using multiple logistic regression. The multivariable logistic regression analysis showed that the possible risk factors of NBM with positive CSF culture in this study were: Length of fever [OR = 1.126; 95% CI (0.999–1.268)], Neurologic symptoms [OR = 3.043; 95% CI (1.164–7.959)], Cerebrospinal fluid protein [OR = 1.001; 95% CI (1.000–1.001)]. Cases of NBM with a longer duration of fever, more neurologic symptoms, and higher levels of CSF protein were more likely to demonstrate positive results on CSF culture.

**Conclusion:**

Cases of NBM with CSF culture-positive results were more likely to have severe clinical manifestations and develop more serious neurologic damage. Patients with NBM who have longer durations of fever, more neurologic symptoms, and higher levels of CSF protein were more likely to have CSF culture-positive results, who should be followed up more closely.

**Key Message**
- Bacterial meningitis is clinically defined as a serious inflammation of meningitis, usually caused by a variety of bacterial infections that may leave sequelae and long-term complications and high mortality rates. Early diagnosis is often difficult, particularly when the patient has been treated with antimicrobials.

## Introduction

Bacterial meningitis is clinically defined as a serious inflammation of the meninges, usually caused by a variety of bacterial infections, and which may leave sequelae and, long-term complications and high mortality rates [1–3]. A cerebrospinal fluid (CSF) culture is considered the gold standard in diagnosing neonatal bacterial meningitis (NBM); however, CSF cultures often have high false-negative rates. Moreover, the clinical manifestations of NBM are non-specific. As such, early diagnosis is often difficult, particularly when the patient has been treated with antimicrobials [4,5].

NBM is an acute bacterial infection of the central nervous system. Early diagnosis plays an important role in guiding definitive antimicrobial management [6]. The diagnosis of NBM is usually based on the CSF culture results, as well as CSF analysis, such as leukocyte count and protein level and using microscopically to assess the presence of bacteria in CSF. Some infections localised in brain tissue could also increase the inflammation, however, it is hard to distinguish in neonates. So far, only a few studies have reported the differences in clinical presentation and outcomes of NBM depending on CSF culture results.

In our study, we analysed the perinatal clinical data, laboratory results, and central nervous system (CNS) imaging studies of patients with NBM and determined whether there was a significant difference between patients with positive and negative CSF culture results. We also examined whether any independent factors increase the likelihood of a positive CSF culture result.

## Materials and methods

### Study participants

This study was a retrospective review of medical records of neonates with NBM. A total of 221 neonates, who were treated for bacterial meningitis in the neonatal intensive care unit (NICU) of Children’s Hospital of Soochow University between January 2015 and May 2019, 35 were abandoned based on exclusion criteria,186 cases were included in our study. Each neonate was treated as per the medical decision made by the assigned attending. We reviewed the demographic features and clinical and laboratory records of each patient. Demographic data included the age at diagnosis, sex, birth weight, gestational age, and maternal conditions such as premature rupture of membrane (PROM), intrapartum fever, chorioamnionitis, and meconium-stained fluids. Clinical characteristics included the presence of any neonatal neurologic symptoms (seizures, hypertonia/hypotonia, fatigue, limb jitter, and staring). Laboratory findings that were collected included complete blood cell count (CBC), blood culture, CSF analysis, C-reactive protein (CRP) as well as hepatic chemistry panel (HCP), glycemic status, and electrolytes.

### Diagnosis of neonatal bacterial meningitis (NBM)

NBM was diagnosed if the clinical signs comply with central nerve system (CNS) infection such as seizures, hypertonia/hypotonia, fatigue, limb jitter, and staring, while fulfil a positive CSF culture or a positive CSF analysis. The positive CSF analysis in term neonates was defined as the leukocyte count >32/mm^3^, protein level >170 mg/dL, and glucose level <34 mg/dl [7]. Similarly, the positive CSF analysis in preterm neonates referred to the leukocyte count >29/mm^3^, protein level >150 mg/dL, and the glucose level <24 mg/dL.

#### Exclusion criteria

**Figure 1.**
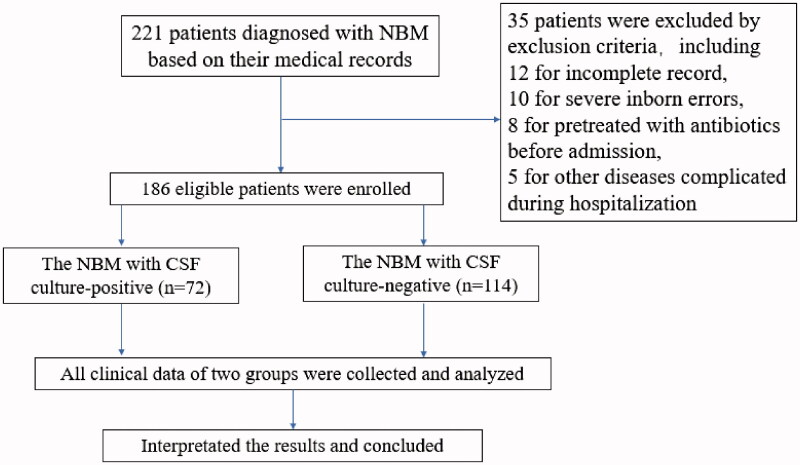
The flow chart of study design.

Neonates who were older than 28 days of age upon diagnosis, evidence of congenital infections, genetic disorders, or chromosomal abnormalities, and neonates who had contaminated CSF samples, the neonates who had been pre-treated with antibiotics before admission, were excluded. The neonates were subsequently grouped based on whether their CSF cultures were positive or negative (Figure 1).

### Ethics statement

All the parents or legal guardians of the participants in this study signed informed consent forms. This study was approved by the Ethics Committee of the Children's Hospital of Soochow University.

### Statistical analysis

Continuous variables are presented as mean ± standard deviation or median ± interquartile range. Differences in categorical variables among the groups were examined using the *χ*^2^ test. Comparisons of continuous variables were analysed using the Mann-Whitney *U* test or *t*-test. Factors were tested through a univariate analysis, and factors with a *p* < .05, were tested in a logistic regression analysis (Forward: LR), adjusting for potential confounding factors (sex, age at onset, birth weight). All tests were two-tailed, and statistical significance was set at *p* < .05. Analyses were performed using SPSS version 22.0 (IBM Corporation, Armonk, NY).

## Results

Among the 186 neonates included in our study, 99 were male and 87 were female. The mean birth weight was 2,977.39 ± 850.61 g. The mean age at onset was 14.97 ± 9.95 days. Seventy-two (39%) cases demonstrated positive CSF cultures, whereas 44 (24%) cases demonstrated positive CSF and blood cultures. Among the positive CSF cultures, *Escherichia coli* was detected in 28 cases (39%); *Group B streptococcus* was detected in 21 cases (28%); *Staphylococcus* was detected in 15 cases (20.8%); *Enterococcus faeces* was detected in 3 cases (4%); and *Listeria monocytogenes, Klebsiella pneumoniae, Pseudomonas aeruginosa, Acinetobacter baumannii,* and *coagulase-negative staphylococci* were each detected in 1 case (1.4% each).

### Baseline characteristics

There were no statistically significant differences in sex, gestational age, and birth weight between the two groups. The CSF culture-positive group demonstrated older ages at onset, higher fever peaks, longer durations of fever, and hospital stay. Meanwhile, the CSF culture-positive group had more frequent neonatal CNS neurologic symptoms (seizures, hypertonia/hypotonia, fatigue, limb jitter, stare), and more frequent imaging abnormalities (subdural collection of fluid, hydrocephalus, brain abscess, cerebral infarction) than the CSF culture-negative group (*p* < .05, Table 1).

**Table 1.** Baseline characteristics in 186 neonates with bacterial meningitis.

Variables	Positive culture	Negative culture	*P*
Sex, male (*n*)	37/72	62/114	.840
Age at onset (days)^a^	16.87 ± 9.11	13.82 ± 10.29	.046
Gestational age (weeks)^a^	38.34 ± 2.83	37.42 ± 3.51	.054
Birth weight (g)^a^	3070.38 ± 942.61	2919.77 ± 787.16	.245
Peak fever (°C)^a^	39.05 ± 0.95	38.56 ± 1.03	.001
Duration of fever (days)^a^	5.59 ± 4.06	3.94 ± 3.53	.004
Maternal factors	10/72	20/114	.544
Neurologic symptoms	26/72	17/114	.001
Hospital stay (days)^a^	37.1 ± 25.8	28.4 ± 14.7	.002
Imaging abnormalities (n)	41/72	43/114	.006

^a^Data are presented as mean ± standard deviation; Maternal factors: premature rupture of membranes, intrapartum fever, amnionitis, meconium contamination; Neurologic symptoms: seizures, hypertonia/hypotonia, fatigue, limb jitter, stare.

### Clinical features and blood and CSF findings

In terms of laboratory parameters, the CSF culture-positive group had higher levels of CRP than the CSF culture-negative group (*p* < .05). There were statistically higher levels of protein, adenosine deaminase, cells, and lower glucose, lactate dehydrogenase of CSF in the CSF culture-positive group than the CSF culture-negative group (Table 2).

**Table 2.** Laboratory values in 186 neonates with bacterial meningitis.

Variables	Positive culture	Negative culture	*P*
Blood parameters^a^			
White blood cell (×10^9^ /L)^a^	11.42 ± 7.30	12.42 ± 7.13	.364
Neutrophil (×10^9^ /L)^a^	6.09 ± 5.53	7.33 ± 5.30	.136
Hemoglobin^a^	125.96 ± 29.85	129.95 ± 24.54	.331
Platelet^a^	283.96 ± 170.71	256.14 ± 150.59	.252
C-reactive protein^b^	35.73 ± 100.00	20.69 ± 81.68	.049
Procalcitonin^b^	2.90 ± 9.14	1.03 ± 6.72	.371
Albumin^a^	34.09 ± 4.16	34.35 ± 4.17	.704
Alanine aminotransferase^b^	13.50 ± 13.05	12.60 ± 14.90	.754
Aspartate aminotransferase^b^	26.25 ± 12.05	30.40 ± 34.00	.053
Prealbumin^a^	78.57 ± 35.92	87.34 ± 40.09	.162
Sodium^a^	134.19 ± 3.47	135.39 ± 4.56	.081
Potassium^a^	4.55 ± 0.57	4.43 ± 0.65	.212
Cerebrospinal fluid (CSF) parameters			
White blood cells (×10^6^ /L)^b^	880.00 ± 3,785.50	90.00 ± 454.00	.001
CSF glucose (mmol/L)^a^	1.62 ± 1.21	2.08 ± 0.88	.007
CSF protein (mg/L)^a^	2355.52 ± 1381.23	1,722.47 ± 1,202.44	.002
CSF adenosine deaminase^b^	4.30 ± 7.00	1.80 ± 3.40	.001
CSF lactate dehydrogenase^a^	417.30 ± 190.28	505.52 ± 360.13	.040

^a^Data are presented as mean ± standard deviation; ^b^Data were presented as median ± interquartile range.

### Independent factors for CSF culture positivity

We also investigated the independent factors that may influence CSF culture positivity among patients with NBM (Table 3). Logistic regression analysis revealed that there were significant statistical differences in the incidence of neonatal neurologic symptoms and CSF protein levels between the two groups. The presence of neurologic symptoms and high protein levels in the CSF were independent predictors for CSF culture positivity among patients with NBM.

**Table 3.** Multivariable logistic regression analyses for prediction of positive culture result^a^.

Variables	B	SE	Wald	*P*	OR (95% CI)
Length of fever	0.118	0.061	3.788	.052	1.126 (0.999–1.268)
Neurologic symptoms	1.113	0.490	5.149	.023	3.043 (1.164–7.959)
Cerebrospinal fluid protein	0.001	0.000	8.219	.004	1.001 (1.000–1.001)
Constant	−6.781	3.144	4.653	.031	0.001

^a^Adjusted for sex, gestational age, and birth weight. Neurologic symptoms: seizures, hypertonia/hypotonia, mental fatigue, limb jitter, and stare; CI, confidence interval; OR, odds ratio; SE, standard error.

## Discussion

NBM is relevant with substantial morbidity and mortality in neonates and requires early diagnosis and therapy [8]. However, despite the availability of antibiotics in most countries, morbidity, and mortality rates remain high among patients with NBM [9,10]. Diagnosing NBM is particularly difficult because diagnosis relies on CSF culture results, which have high false-negative rates [11,12]. As such, many studies have proposed to diagnose meningitis based on the combination of clinical signs, positive blood cultures, and abnormal cerebrospinal fluid parameters instead. We used these same diagnostic criteria in our study [13–15]. We also analysed whether positive CSF culture results could be predicted by independent factors, such as the length of fever, presence of neurologic symptoms, and amount of protein in CSF.

In our study, we found that CSF culture-positive NBM had higher levels of CSF protein than CSF culture-negative NBM. In their study, Tan et al. [16] reported that neonates with NBM who had poorer outcomes also had higher levels of CSF protein. This indicates that high CSF protein levels in neonates with NBM may be a good marker for more aggressive therapy. High CSF protein levels may also identify patients who are good candidates for new treatment strategies, with the aim to reduce near-term complications of NBM. Our study also showed that the CSF culture-positive group had higher levels of WBCs and ADA in their CSF samples than the CSF culture-negative group.

Neonatal neurological symptoms, such as seizure, hypertonia/hypotonia, fatigue, limb jitter, and stare, were more likely to be observed in the CSF culture-positive group. Lee et al. [17] used the bacterial meningitis score system to investigate the features of bacterial meningitis and found that patients with high scores more likely presented with seizures. Klinger et al. [18] found that seizure duration >72 h and coma were the key predictors of adverse outcomes in NBM. These studies suggest that patients with CSF culture-positive results are more likely to have serious neurologic damage compared to patients with CSF culture-negative results.

In our study, we found that CSF culture-positive neonates had a longer hospital stay and more imaging abnormalities, so we think the CSF culture-positive neonates would have more short-term complications. We tried to find the risks for CSF culture positive, which also would be regarded as the risks for neurologic damage in NBM. So the patients who had those risk factors could achieve early aggressive therapy.

CSF culture-positive neonates also demonstrated significantly high levels of CSF protein, which reflects an ongoing inflammatory response. During the acute phase of NBM, increased interstitial vascular permeability allows pathogens to cross the blood-brain barrier. These pathogens release inflammatory mediators, such as teichoic acid and endotoxins, which, in turn, incite an immune response. The increase in inflammatory mediators, such as polymorphonuclear leukocytes, interleukins, and cytokines, in the CSF presents an increased CSF protein count on CSF analysis. However, the same immune response also results in the formation of reactive oxygen species. These free radicals are highly unstable and may produce harmful proteins, lipids, and nucleic acids. High lipid levels in the setting of low cerebral antioxidant levels are harmful to the central nervous system [19,20]. As such, while the inflammatory cascade in NBM aims to protect the central nervous system, it is also the cause behind neurologic morbidity.

Our study also found that *Escherichia coli* was the most prevalent pathogen in NBM at our centre. This was similar to the results reported in many other developing countries [3]. Another prevalent bacterial species was *Group B streptococcus*. Identifying the most common causative agents may help us better understand the epidemiology of NBM, as well as allow us to make more appropriate empirical decisions.

A significant problem, particularly in China, relates to the high utilisation rate of antimicrobial drugs. Patients have often been prescribed antibiotics at the outpatient level, which increases the likelihood that CSF cultures produce false-negative results [21]. Some researchers have found that antibiotic treatment prior to admission reduces CSF culture positivity rates by nearly 30% [22,23]. We did not record the number of neonates who received antibiotic treatment prior to admission in our study; therefore, further research is needed to confirm whether such a correlation exists.

Our analysis also has other limitations. First, our study was a retrospective study and may have suffered from selection bias. Second, all our data were collected from a single centre, resulting in a relatively small sample size. Examining NBM cases across multiple centres and with a larger sample size may provide more generalisable results.

## Conclusions

Patients with NBM with CSF culture-positive results are more likely to present with severe clinical manifestations, as well as develop serious neurologic damage. Patients with NBM who present with neurologic symptoms or have higher levels of CSF protein should be followed-up more closely.

## Data Availability

The data that support the findings of this study are available on request from the corresponding author, Xueping Zhu. The data are not publicly available due to their containing information that could compromise the privacy of research participants.
